# Application of a Flow-Induced Stress Wave and Investigation of Associated Injuries on Cell Monolayers Using a Parallel Plate Flow Chamber

**DOI:** 10.3390/mps3040065

**Published:** 2020-09-24

**Authors:** Samar Shurbaji, Mahmoud Khatib A. A. Al-Ruweidi, Fatma Hassan Ali, Fatiha M. Benslimane, Huseyin C. Yalcin

**Affiliations:** Biomedical Research Center, Qatar University, Doha, P.O. Box 2713, Qatar; ss1104227@qu.edu.qa (S.S.); ma1207471@student.qu.edu.qa (M.K.A.A.A.-R.); fa1300703@student.qu.edu.qa (F.H.A.); fatiha@qu.edu.qa (F.M.B.)

**Keywords:** flow, stress, parallel plate flow chamber, cell injuries, epithelial cells, endothelial cells, viability, hemodynamics, bubble

## Abstract

Parallel plate flow chambers are widely used to expose cultured cells to physiological flows for the investigation of a variety of diseases. These applications usually involve the generation of continuous and steady fluid flow over cell monolayers for extended durations, usually a few days. Another technique is to generate a fast high-stress wave over the cells to see the immediate effect of flow-induced stresses. This can be achieved by propagating an air/liquid interface, in other words, a bubble, over cell monolayers. The approach is relevant to the reopening event of fluid-filled lung bronchioles and alveoli during mechanical ventilation therapy of Acute Respiratory Distress Syndrome. This article explains how we generate a stress wave using a parallel plate flow chamber and presents representative results of this wave on cultured lung epithelial cells.

## 1. Introduction

Cells in the human body are constantly exposed to physical forces [1]. These forces can be defined as stress (i.e., force per unit area). Shear stress is the frictional force exerted on a solid surface by the flowing fluid over the surface, and normal stress is the perpendicular pressure force on the surface. In the human body, these forces can act on cells lining the walls of the vessels, such as endothelial cells (ECs) [2] in blood vessels and epithelial cells (EpCs) [3] in pulmonary vessels. While physiological levels of these stresses are important in proper cell functioning, abnormal levels were shown to implicate the progression of diseases due to cellular injuries [4,5]. In most cases, these diseases are studied in vitro by exposing cultured cells to flow-induced stresses. This can be achieved by the use of parallel plate flow chambers, in which cultured cells in the bottom coverslip are exposed to fluid flow. In most such applications, cultured cells are exposed to continuous steady flow for an extended duration. An alternative approach is to expose these cells to a fast-high stress wave to see the immediate effect of flow-induced stresses on these cells. This high-stress wave can be generated by propagating an air/liquid interface, in other words, an air bubble, over cell monolayers.

This approach is most relevant to the investigation of Acute Respiratory Distress Syndrome (ARDS). ARDS results in hypoxemia due to increased permeability of the alveolar/capillary barrier at the distal lung [6]. This leads to the accumulation of edema fluid within gas exchange sites. Moreover, in ARDS, the surface tension in the lung is increased because the surfactant molecules in the thin liquid layer over epithelial cells are inactivated, making delicate lung tissue prone to injury [7]. ARDS patients need mechanical ventilation in order to inflate their lungs. However, mechanical ventilation causes more damage to delicate epithelium linings of alveoli and small airways due to the enforced displacement of edema liquid, a phenomenon known as Ventilator-Induced Lung Injury (VILI) [8]. The VILI is a result of air bubble propagation during airway reopening. 

The recent COVID-19 virus that has been classified as pandemic is characterized with a second outcome of an acute respiratory illness in many patients (clinical characteristics) [9,10]. This condition has been classified as Corona Acute Respiratory Distress Syndrome (CARDS) [11]. The CARDS’ severity is no different than the ARDS’, the patients would need their lungs to be inflated mechanically in many cases [12]. It appears that the mortality rate is higher in ICU (Intensive Care Unite) patients who needed mechanical ventilation, according to reports from China and the USA [12,13]. The mortality rates in ICU COVID-19 mechanically ventilated patients could be not only due to COVID-19 complications, but could also be due to injuries caused by VILI as well. Therefore, methods and protocols need be to developed to study the effects of shear stress on lungs caused by mechanical ventilations.

**Figure 1:**
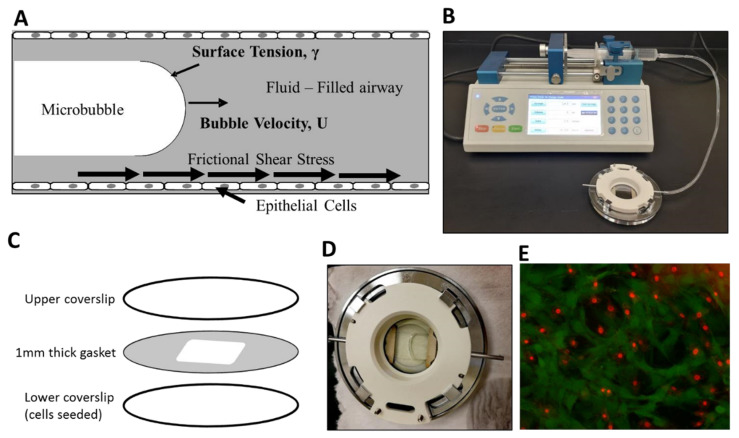
Experimental setup. (**A**) We exposed epithelial cells to frictional shear stress relevant to airway reopening in our setup. (**B**) A single bubble is propagated over cell monolayer by filling the chamber and then retracting fluid over the cells. (**C**) The parallel plate flow chamber is composed of an upper cover slip, a cell-seeded lower coverslip, and a membrane sandwiched between two coverslips. (**D**) A propagating bubble is seen. (**E**) Fluorescent live/dead stain method is used to assess viability. Here, green represents calcein-stained live cells, and red are ethidium-stained dead cells.

For better understanding the effects of bubble flow-induced damage caused by mechanical ventilation, we have previously developed an in vitro model of airway reopening [14,15]. In the developed model, the simulation of reopening dynamics was manifested through air bubble propagations inside an adjustable height parallel plate flow chamber, as shown in Figure 1. Here, bubble propagation was created with a syringe pump, and a variety of fluids can be tested on confluent or sub-confluent monolayers of EpCs. For the calculation of levels of normal and shear stresses in such a configuration, please refer to our previous studies [14,15]. In this paper, we present our protocol for the generation of a bubble flow-induced stress wave on cell monolayers. The parallel plate chamber is composed of a bottom cover slip, an upper coverslip, and a silicone membrane sandwiched between the coverslips. Cells are cultured on the bottom coverslip, and the cell-seeded coverslip is transferred to the flow chamber prior to bubble propagation. Through connecting a syringe pump to the system and filling and retracting fluid over the cell monolayer, bubble propagation is achieved. Following the bubble propagation, cellular injury is assessed by conventional live/dead assays based on fluorescent microscopy.

## 2. Procedure

### 2.1. Cell Culture

1. Coating 40 mm Diameter Coverslips with Collagen
  Note: 40 mm coverslips have to be used as they are compatible with the apparatus (Bioptechs FCS2 Flow Chamber). These are the bottom coverslips that we seed the cells on.
2. Coverslips’ Preparation
  1. Coverslips can be sterilized via different sterilization methods; here, we used autoclaving at 121 °C for 30 min.
    Note: The steps below have to be performed in a biological hood under sterilized conditions to prevent contamination.
  2. Prepare (25 µL/mL) collagen solution using Collagen I Rat Protein, Tail (ThermoFisher scientific, CAT# A1048301). The stock solution is 3 mg/mL, and to prepare a 25 µL/mL collagen solution, dilute 416 µL collagen solution in 50 mL distilled autoclaved water.
  3. Place the cover slips in individual well plates (here, we used 60 mm diameter wells to fit 40 mm coverslips) and add a total of 2 mL of the coating solution to each well. Cover the plates and leave the coverslips to incubate with the collagen coating solution for 1 h at room temperature.
    Note: Collagen coating is critical as cells might detach while being exposed to bubble flow-induced stresses in the flow chamber, so, it is necessary to prevent such detachment. Other matrices, like poly-L-lysine or fibronectin, can be used in case of unavailability of collagen.
  4. Wash coverslips with 1 mL of PBS (Phosphate Buffer saline) and allow them to air dry (around 15 min).
3. Seeding Epithelial Cells on Coverslips

### 2.2. Harvesting

**Figure 2:**
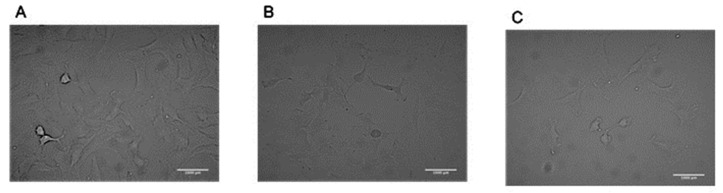
149 lung epithelial cells imaged with a 20X objective (**A**) ~80% confluent, (**B**) ~50% confluent, and (**C**) ~30% confluent.

1. To harvest A549 cells at a density of 5 × 10^4^ cells/mL and 149 L2 cells at a density of 3 × 10^4^ cells/mL, the following protocols should be followed.
  Note: This protocol is for A549 (ATCC CCL-185) and L2 (ATCC CCL-149) lung epithelial cells. A549 cells are accepted as type-2 alveolar epithelial cells, whereas L2 cells are accepted as type-1 [11]. Type-1 cells comprise ~95% of the alveolar surface area and function mainly in gas exchange, whereas type-2 cells make up ~5% of the alveolar surface area and are necessary for surfactant cytokine synthesis. To culture A549 or L2 cells, ATCC recommends Ham’s F12K media (Thermofisher scientific, CAT# 21127022) supplemented with 10% fetal bovine serum (FBS) and 1% antibiotic and antifungal solution (Thermofisher scientific, Cat 15240062).
  For cells stored in liquid nitrogen:
  1. Take the cells (cryotube) from liquid nitrogen and allow them to thaw.
  2. Add pre-warmed 1 mL media to the cryotube and transfer it all to a 15 mL tube.
  3. Repeat step 2 for two to three times to assure all cells have been transferred.
  4. Centrifuge the tube at 16.1 g for 5 min.
  5. Discard the supernatant and keep the pellet.
  6. Re-suspend the pallet in 1 mL media.
  7. Prepare two T25 flasks for each thawed cryotube. Add 5 mL media to each flask.
  8. Add 500 µL from the cell suspension to each T25 culture flask (Thermofisher scientific, Cat 156367).
    Note: The amounts of cells added will depend on the number of cells stored. The seeding density of both 149L2 and A549 should be cells/mL in a T25 flask.
  9. Incubate the flasks in a 37 °C incubator supplemented with 5% CO_2_ and 95% air until they become confluent.
    Note: The media needs to be changed every 2–3 days.
  For confluent cells grown in a T25 culture flask:
  1. Discard the media, then wash the cells twice with pre-warmed 5 mL PBS (wait around 30 s in between each wash).
  2. Add 1–2 mL of 0.25% trypsin solution (Thermofisher scientific, Cat 25200056) and incubate the cells in the incubator for 5 min.
  3. Check the cells under the microscope for cell detachment, if the cells are floating (detached), add 5 mL media (with FBS) and transfer the media with cells to a 15 mL tube.
  4. Centrifuge the tube at 16.1 g for 5 min.
  5. Discard the supernatant and keep the pellet.
    Note: If you are using a T75 flask, the same protocol is applied but with 3 mL 0.25% trypsin solution, and add 7 mL media after cell detachment (in step 3).
2. After harvesting, re-suspend the pellet in 1 mL of pre-warmed complete media.
3. Count the number of cells needed to become confluent (using a trypan blue-hemocytometer or automated counting).
  For manual counting using a hemocytometer:
  1. Add 80 µL of trypan blue to a 1.5 mL Eppendorf tube and 20 µL of the cell suspension and mix well.
  2. Place a coverslip on the top of the hemocytometer slide.
  3. Insert 10 µL of the suspension to one side of the hemocytometer (the suspension is added inside the groove).
    Note: For more accurate counting, the suspension is loaded into both sides of the hemocytometer, thus eight gridded areas are counted.
  4. Count live cells of four gridded areas under the microscope and record an average number of cells for the four areas.
    Note: Live cells appear non-blue when stained with trypan blue, as trypan blue gets into dead cells and stains them. Only live cells are counted.
  5. To find the concentration of cells suspended in the cell/mL unit, use Equation (1).
    Note: For CCL-149 (known as L2) rat lung cells, a concentration of 8 × 10^4^ cells/mL and 2 mL cell media can be used for the cells to become confluent in four days. For CCL-185 (known as A549) cells, a concentration of 1.4 × 10^4^ cells/cm^2^ with 2 mL media is used for the cells to become confluent in four days.
    Cell concentration (cell/mL) = Average # of cells counted in 4 or 8 areas × dilution factor × 10^4^ (1)
    where the dilution factor in our case is 5, and 10^4^ is the hemocytometer constant.
4. Place sterilized 40 mm collagen-coated coverslips on 50 mm Petri dishes.
5. Add media to the petri dish and then add the required number of cells.
  Note: Homogenous cell distribution is critical; to avoid cells growing in one area only, the dishes must be shaken, or the cells must be mixed well with the media.
6. Incubate flasks at optimum growth conditions (5% CO_2_, 95% air, and at 37 °C).
7. Change the media every 2–3 days and wait until cells are ≈80–100% confluent. (Figure 2)

### 2.3. In Vitro Flow Chamber and Bubble Propagation over Cell Monolayers

#### 2.3.1. Flow Chamber Assembly and Flow Rate Calculations

##### Parallel Plate Flow Chamber Assembling

**Figure 3:**
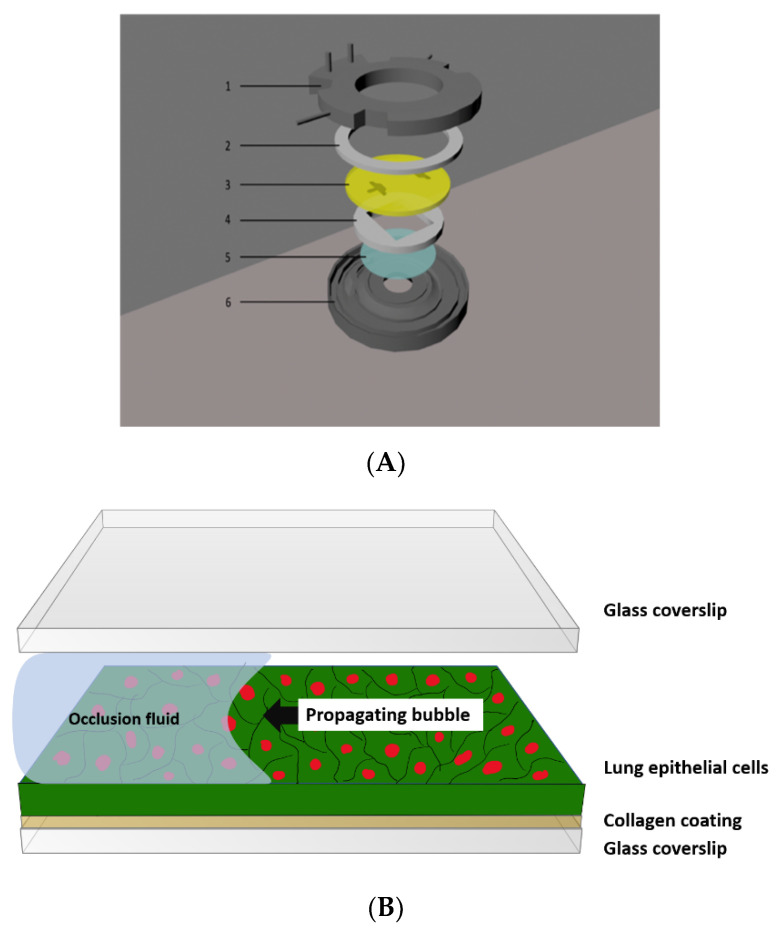
(**A**) The FC2 flow chamber consists of: (1) the upper half (top), containing inlet and outlet perfusion tubes, (2) an upper gasket, (3) a microaqueduct slide, (4) a singular lower gasket, 1 mm thick, (5) a 40 mm coverslip, and (6) a self-locking base. (**B**) Assembled flow chamber.

The BIOPTECHS FCS2 flow chamber is made of six different parts: the lower aluminum part, the upper plastic part (has two inlets), two silicon gaskets, one slide, and a coverslip insert where the coverslip containing cells can be placed (Figure 3).

1. To assemble the flow chamber, an upper, round, 30 mm hole gasket is placed under the upper half containing perfusion tubes (inlet and outlet tubes).
2. Followed by a microaqueduct slide, a singular, lower, 14 × 22 mm rectangle gasket, a 40 mm cell-seeded lower cover glass, and a self-locking base are assembled.
3. The upper half perfusion inlet tube is connected to the syringe pump to apply flow perfusion over the cells.

##### Flow Rate Calculation

To find the flow rate (velocity of 0.3 mm/s), use Equation (2).

Note: This velocity was selected as it corresponds to the velocity that is expected to be found in the terminal and respiratory bronchioles [12]. A gasket of 1 mm thick is selected to represent the airway’s diameter.

(2) Q=U×A

where *Q* is the flow rate, *U* is the velocity, and *A* is the cross-sectional area of the flow channel.

Note: For this configuration (gasket width of 13 mm and gasket height of 1 mm), *A* is calculated as 13 × 1 = 13 mm^2^. To get the desired bubble velocity of 0.3 mm/s, flow rate *Q* is 3.9 mm^3^/s.

#### 2.3.2. Connecting the Syringe Pump to the Flow Chamber

1. The upper white part has an inlet and outlet: connect one of the ends to 1/16” Tygon Tubing.
2. Fill a syringe with warm phosphate buffered saline (PBS), which represents a high surface tension fluid, a characteristic of lung edema in ARDS.
3. Place the syringe onto a syringe pump and connect the syringe to the flow chamber. Here, we used a CHEMYX model fusion 720 syringe pump.
4. Turn on the syringe pump and set the syringe volume that is used in the experiment (syringe volume can be selected from built in selection with syringe brand, available in most syringe pumps).
5. Set volume and flow rate.
6. Press ‘’infuse” and then press “start”. Wait for the PBS to exit the flow chamber from its outlet. Previously, we showed that this initial perfusion does not cause any cell injury [15,16,17,18].
7. Once PBS comes out from the outlet, press “stop” and select “withdraw” so a bubble starts to propagate over the cells, exposing cells to bubble flow-induced stresses.
  Note: The fluid will be completely withdrawn from the chamber in a few seconds. The coverslip should be immediately placed in cell media or stained to prevent cell dryness.
8. If needed, bubble propagation can be repeated multiple times to simulate multiple reopening events.
9. When all PBS is withdrawn, stop the machine and disassemble the chamber (make sure when removing the coverslip that cells are facing upwards) to transfer the cover slip to a well for live/dead stain analysis. Alternatively, without disassembly, live/dead stain can be perfused to the chamber to perform cell staining in the chamber.

### 2.4. Quantification of the Cellular Injury after Bubble Propagations

**Figure 4:**
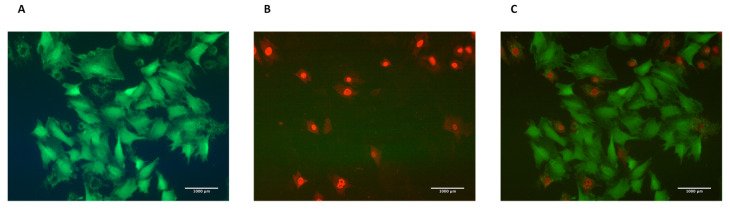
(**A**) Live cells stained with calcein stain visualized using the GFP filter. (**B**) Dead cells stained with ethidium homodimer-1 stain visualized using the CY3 filter. (**C**) Merged picture of live and dead cells using ImageJ software.

1. A conventional fluorescent live/dead stain kit from Thermo Fisher Scientific (Cat. No. L3224) is used to quantify the viability. For this assay, m/2000 dilution is made for calcein-AM (for identification of live cells) and ethidium homodimer 1 (for identification of dead cells), with a final concentration of 1 µM, in the serum-free media [13].
2. Transfer the coverslip to a 50 mm petri dish and add ≈1–2 mL of the stain to the coverslip. The stain is light-sensitive so the plate containing the coverslip should be covered with aluminum foil.
3. Incubate for 15 min at 37 °C.
  Note: Cells can be kept at room temperature if needed for this stain. We have shown that keeping the cells at room temperature for 15 min does not induce any additional cell death [12].
4. Visualize the slide under a fluorescent microscope (GFP (Green Fluorescent Protein) filter for live cells and CY3 filter for dead cells). Figure 4 represents cells stained with Live/Dead assay.
5. Take 5–10 pictures for different fields. We usually use 10X or 20X objective to have a field of about 400–500 or 80–200 cells, respectively.
  Note: Fluorescent pictures should be taken from the middle portion of the channel to eliminate effects of walls on the sides. We have realized that more cells die in the regions close to side walls. Also, cell confluency is an important factor in cell injury for this type of perfusion experiment [14]. Therefore, to compare different experimental groups, it is advised to culture cells to the same confluency level.
  Note: For an identical field, one live image (green filter) and one dead image (red filter) are taken. These images are then merged to visualize live and dead cells in the same image for the studied field. Image acquisition software can be used for merging. Alternatively, ImageJ can be used for this step.

#### 2.4.1. To Merge Images Using ImageJ

1. Open the images (File → open → select the images).
2. Change the images to 8-bit format (image → type→ 8-bit).
3. Select image → color → merge channels.
4. Choose the image name based on its color (dead cells’ image will be in the C1 (Channel1) and live cells’ image in C2 (Channel2)).
5. Select “create a composite” → OK.
6. Change the image to RGB (Red,Green,Blue) format (image → type → RGB color).

#### 2.4.2. Counting Dead and Live Cells to Quantify Viability

1. The total numbers of live and dead cells need to be determined to quantify cellular injury. A variety of software applications such as Metamorph, Image pro plus, and ImageJ can be used in an automated or manual way. We used ImageJ for this purpose
2. Open ImageJ → File → open → select the photo → go to plugins → analyze → cell counter → select type 1 and start counting → results.
3. To find the viability rate, use Equation (3):
  (3) $$Viability\;rate\;=\frac{Number\;of\;dead\;cells\;}{Total\;number\;of\;cells}\times 100$$

## 3. Expected Results

After seeding cells and allowing them to grow until they are confluent, cell-seeded coverslips were randomly divided into two groups: the first group is the control group (cells are not subjected to any stress using the flow chamber, only washed with PBS and stained using live/dead staining), while the other group is an experimental group (cells were subjected to bubble flow-induced stresses in the flow chamber, then stained with live/dead staining). 

After visualizing both the groups under a fluorescent microscope, images were taken and merged using ImageJ to quantify the viability rate for each experimental run. Control groups showed a death percentage of around 0–0.2%, whereas the bubble stress group showed a death percentage of around 53%. This difference is highly significant when compared to control groups using a t-test, with a *p*-value of less than 0.01.

**Figure 5:**
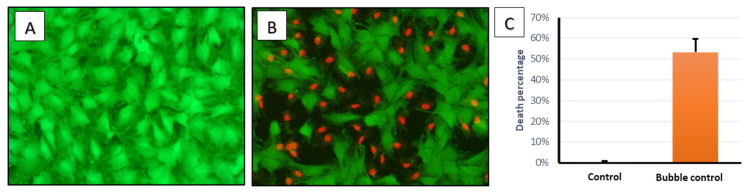
Fluorescent images for 149 L2 cells: (**A**) control group, and (**B**) experimental group, where a bubble was formed to induce a stress wave on the cells. Green color is for live cells while red color is for dead cells. (**C**) Bar chart for control and experimental group, showing a significant difference (*p*-value < 0.01) in death percentage. Error bars are for standard error.

This experiment was repeated seven times for both groups, showing consistent results in all replications. In Figure 5 below, we present average and SE values. 

### Effect of Mechanical Stress on Cytoskeletal Structure 

**Figure 6:**
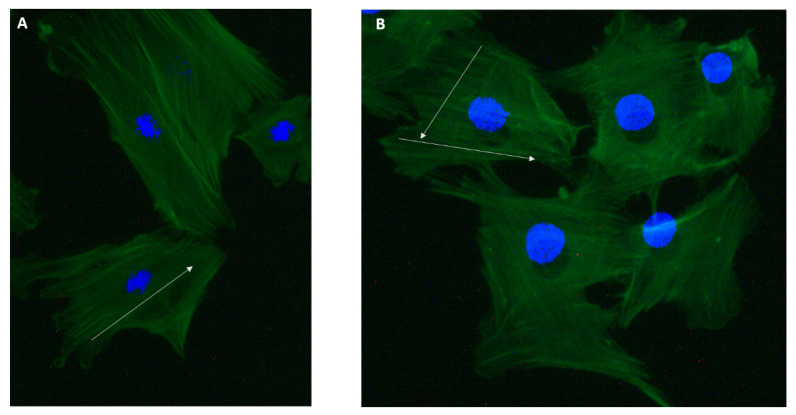
(**A**) Cell’s cytoskeleton for the control group, and (**B**) cell’s cytoskeleton for the bubble-propagated group. The green color represents actin filaments, and blue is the nucleus stained with DAPI (4’,6-Diamidino-2-Phenylindole). The white arrow represents the direction of actin filaments’ orientation.

Mechanical stress can change the cell’s cytoskeleton structure and actin filaments’ formation. Here, we stained cells after bubble propagation to see the changes in the cytoskeleton structure. Actin filaments were stained with actin-labeled Phalloidin. Figure 6 shows the cytoskeleton of 149 L2 cells for control and bubble-propagated samples. Single bubble propagation did not show a significant difference in cytoskeletal structure. However, slight differences in actin filaments’ orientation were shown. Actin filaments were oriented in two directions in the bubble-propagated sample (Figure 6B), whereas in one direction in the control group (Figure 6A).

## 4. Conclusions

Physical stresses can be applied to cultured cells using different methods. In this protocol, we demonstrated the application of a fast and transient stress wave on cultured cells using a perfusion chamber. This method is relevant to airway reopening of fluid-filled lungs for ARDS. Using this technique, the influence of mechanical stress on cells can be studied. Also, approaches to decrease cellular injuries for clinically relevant mechanical stresses, such as pre-exposure of cells to tested agents, can be studied.
